# Briefing Note: Proceedings of the Singapore National Clinical Pharmacy Colloquium—Health Manpower Development Planning

**DOI:** 10.3390/pharmacy6020054

**Published:** 2018-06-12

**Authors:** Richard H. Parrish, Lita Chew

**Affiliations:** 1 St. Christopher’s Hospital for Children, American Academic Health System, Philadelphia, PA 19134, USA; 2 School of Pharmacy, Virginia Commonwealth University, Richmond, VA 23298, USA; 3 Department of Pharmacy, National University of Singapore, Singapore 117543, Singapore; Lita_CHEW@moh.gov.sg; 4 Ministry of Health, Pharmacy Services, Government of Singapore, Singapore 117543, Singapore

**Keywords:** clinical pharmacy, human resources, Singapore, non-medical prescribing, practice standards

## Abstract

The nation of Singapore highly values its health service as well as the component of healthcare delivery that includes clinical pharmacy and administration. The Ministry of Health (MOH) engaged the services of Dr. Richard Parrish to better understand the relationship between the Singaporean citizenry and its clinical pharmacists. Through a series of five lectures, structured from the national to local unit level, four roundtables, two hands-on sessions, and three workshops, clinical pharmacists and administrative leadership participated in “open-mic” style lectures, discussed the issues facing Singapore pharmacy in its current provision of healthcare services, and deliberated on the future resources required to meet projected healthcare needs. From three distinct perspectives, these discussions were very frank, transparent, and passionate about what practitioners and administrators thought Singaporean health leadership needed to address in terms of (1) what programs and practices to continue; (2) what ones to start; and (3) what ones to evaluate. Each of the areas below will be framed using these three perspectives in order to clearly reflect the ideas and suggestions expressed in each of the 14 group sessions. These recommendations are forwarded to MOH pharmacy leadership for consideration and action. In addition, we met with MOH leaders regarding non-medical prescribing (NMP), and discussed strategies and tactics that seemed successful as well as unsuccessful in other jurisdictions when adopting expanded scope models for clinical pharmacists and other qualified healthcare providers that include prescribing of medications.

## 1. Key Strengths of Clinical Pharmacy and Administration in Singapore

Singapore, in general, and healthcare and clinical pharmacy, in particular, are rich in cultural diversity. Because there are four official languages, Singapore pharmacy has a number of strengths that have developed from this diversity, coincident to the rise of clinical pharmacy, internationally. The 5.7 million people in Singapore have a relatively high life expectancy and benefit from a robust healthcare system that includes public and private financing schemes as well as an extensive array of institutional, transitional, and ambulatory care facilities. Three large corporate chains own most of Singapore’s 150 community pharmacies, where over 300 pharmacists are employed. Healthcare provision is structured from the national to unit level where clear divisions of scope and service administration are evident. The Pharmacy Practice Act [1] was revised in 2008, and the Singapore Pharmacy Council (SPC) recently completed work on the competency framework that has identified advanced practice roles for pharmacists in areas of: (1) cardiology; (2) oncology/haematology; (3) infectious diseases; (4) neurology/psychiatry; and (5) nutrition support. These advanced practice professionals are certified through attainment of board certifications offered through the American Board of Pharmacy Specialties affiliated with the American Pharmacists Association. Current legislation is pending in parliament that will recognize the signatures of advanced practice professionals for governmental reimbursement and subvention. Moreover, the government recently sub-divided the country into three health districts: (1) west; (2) central; and (3) east. The Health Sciences Authority, an United States Food and Drug Administration (US-FDA) equivalent agency, utilizes the World Health Organization (WHO) essential drugs list as a basis for the provision and authorization of drug products in the country, much of which is imported from China, India, and Australia. The Ministry of Health (MOH) also has developed a structure for advocating for integrated health practices. Within this framework, the Agency for Integrated Health includes the Integrated Health Information System (IHIS). IHIS currently is being leveraged to uncover opportunities for creating point-of-care functionalities and processes that capture discrete episodes of direct care that clinical pharmacists provide. The MOH is well aware of international trends in pharmacist care provision, and has identified an opportunity to expand advanced generalist practice through creation of post-graduate year 1 and year 2 (PGY1 and PGY2) pharmacy practice residencies. In the public sector, a unified informatics and automation strategy called the National Health Integrated Pharmacy System (NHIPS) attempts to dovetail into the National Electronic Health Record in areas of adherence to medications, ordering of medications, and caregiver support tools. These functionalities are accessible through the Health Portal. This consolidated approach has broad appeal to many in the MOH and wider professional constituencies.

Within the largest hospital, Singapore General Hospital, (SGH) an impressive level of automation and technology is operative, improving service line efficiencies in cancer, dermal, dental, neurosciences, ophthalmologic, and cardiac services. In addition, SGH pharmacy uses an integrated discharge medication service to complement inpatient services. The range of services requires that 450 full-time equivalent (FTE) pharmacy personnel are needed; a career ladder framework-basic, intermediate, and advanced practitioner-is established to promote internal advancement and job satisfaction. These ladders also include pharmacy technicians, which comprise 50% of the pharmacy workforce. Although not regulated through the SPC, technicians are seen as important contributors to overall departmental success, especially in the production of parenteral products, such as intravenous hazardous drug compounding and hyperalimentation, as well as non-sterile drug compounding. The MOH uses the GS1 standard for medication dose bar coding. A video produced through SGH pharmacy demonstrates the pharmacy service desire to provide patient-centric, collaborative care. SGH has a well-developed Drug Information Service. Specialties and sub-specialties in pharmacy developed from a variety of needs and wants, both internal and external to the profession. These service functions include: Medication Adherence, Medication Optimization, and Patient Wellness. While these service functions could be argued as components of the pharmaceutical care philosophy of practice, much of the approach for measuring outcomes is based on cost avoidance. Revenue streams include block funding to the newly-designated private limited scheme applied to hospitals, private third party insurances, and patient copays.

Most, if not all, of the clinical work that pharmacists undertake is not directly compensated. Of the 2700 pharmacists licensed under SPC, 2400 practice in the public sector. The other 300 pharmacists practice in private settings, like community pharmacies. In the public sector, pharmacists are not directly compensated for any aspect of care, unlike their private counterparts, who receive medication and service fees bundled into reimbursements for services rendered.

Entry into the health system usually starts with the polyclinic visit, some of which have medications available, and most that do not include pharmacists in any tangible capacity. MOH is directing the change in focus of care from institutional to community or home. In addition, it is shifting reimbursement from quality to value, and healthcare to health. These changes in focus have set in motion a number of local disease management functions that could be performed by any willing and qualified provider, including clinical pharmacists and advanced practice nurses.

*Activities to continue*

- *Integrated approach to care provision*
- *Work on competency framework to include curriculum reformation, objective measurement of competency at entry to practice, and post-graduate educational development*
- *Development of technician practice that complements clinical pharmacist role*
- *Further development of advanced practice or full scope practice for pharmacists*

*Activities to start*

- *Implementation of non-medical prescribing that emphasizes efficiency, quality, and affordability*
- *Determination of ‘cost to dispense’ and ‘cost to advise’ for clinical pharmacy portion, in both private and public scenarios*
- *Develop a pediatric formulary based on World Health Organization (WHO) essential drug list for children*
- *Develop a separate medication management system for children, both in institutional and ambulatory care*
- *Standardize the nomenclature for pharmacy practice work in terms of Resource-based Value Units (RBVUs), to reflect clinical and production/distribution productivity.*
- *Authorize the credentials—e.g., Board Certified Pharmacotherapy Specialist (BCPS) and Board Certified Ambulatory Care Pharmacist (BCACP)—as generalist certifications for advanced practice in pharmacy*

*Activities to evaulate*

- *Attempting to forward value for money arguments and allocations without a determination of the ‘cost to advise’ and the ‘willingness to pay for services’ derived from third parties, government, and patients*
- *Undervaluing the work of the pharmacy technician in support of pharmacy practice due to the lack of a peer regulatory structure*
- *Implementing a supplementary/dependent prescribing role of pharmacists, nationally and generally, through concerns that many pharmacists cannot be expected to prescribe with any level of quality, requiring an assessment and demonstration of continual competency for prescribing undertaken through SPC*

## 2. Key Challenges Facing Clinical Pharmacy and Administration in Singapore

A number of challenges for clinical pharmacy and administration exist in the environment of care delivery. These challenges are summarized in the three themes that permeated the colloquium: (1) ‘what got pharmacy here (present practices from the past), will not get pharmacy there (future); (2) ‘define (your practice) or be defined’ (by others); and (3) Singapore problems require Singapore resolutions. Realization that how we describe pharmacy practice is best performed within the community of pharmacy. A number of participants present at the colloquium have different conceptions of pharmacy practice based on culture, history, and traditions that influence perceptions. This tendency was illustrated in the entrepreneur session where (1) a mini-lecture about entrepreneurship and business planning was given and (2) a game in which four teams were given a rewards structure, whose goal was determined through team interaction and voting, was played. The nomenclature of business is not widely known within the public sector pharmacy community. The tradition of subvention for pharmacist salaries and benefits in the public sector is different than that of the private sector, although there was no representation of community or corporate pharmacy at the colloquium. In addition, there was no representation of any other health profession or governmental agency (other than the chief pharmacist’s office). Therefore, one key challenge is deriving an advisement process that brings together all stakeholders in system transformation to integrate non-medical prescribing. This is no easy task. However, consultation is required in order to appreciate the promises and perils of success. A joint advisory board made up of all medical and non-medical prescribers is suggested to create shared standards for medication prescribing and advisement. Moreover, there are cultural impacts that influence care provision as well; it is uncertain whether the cultural makeup of Singapore was fully represented at the colloquium. The greatest challenge to Singapore pharmacy in the next 50 years is to fully integrate pharmacy technicians and community pharmacy practices (including Chinese, Indian, Malay, and indigenous traditions). Further, Singapore is challenged through a reliance on westernized medicine and pharmacy practices while many indigenous traditions continue to be practiced. Singapore needs to develop its own unique resolutions to population health problems through programs aimed at responsive care practices based on predictive analytics. Otherwise, optimization of the benefit of medications will not be within the reach of any Singaporean.

*Action to continue*

- *Dialogue with corporate pharmacy leaders about the impacts of practice change directives on community practice*
- *Search for unique resolutions to Singaporean pharmacy problems at the structural, process, and outcome levels of analysis*
- *Dialogue about how to transform education, post-graduate training, and advanced practice credentialing within a framework of inclusion of all pharmacy-related personnel*

*Actions to start*

- *Creation of a joint advisory board that encompasses all providers that have prescribing as a function of their practice mission*
- *Dialogue about changes to pharmacist compensation strategy in both public and private sectors with a basis of Relative Value Units (RVUs) [2,3,4]*
- *Incorporate pharmacy technicians within the regulatory framework of pharmacy practice*

*Actions to evaluate*

- *Communicating pharmacy’s value in terms of clinical outcome rather than cost avoidance or cost savings*
- *Alignment of only the public sector to discussions of key performance indicators or core measures of pharmacy value*
- *Portraying pharmacy practice in a silo-ed fashion (i.e., specialty, sub-specialty; public and private)*

## 3. Recommendations to Improve the Practice Delivery of Clinical Pharmacy and Administration in Singapore

While many pharmacists are practicing at full scope, pharmacy practice and administration in Singapore can be improved through implementation of a core measures (CM) or key performance indicator (KPI) program, designed for the unique needs of Singapore. Discussions on these concepts were centered on enumeration of common measurement needs and objectives expressed by participants. These objectives were based on two core principles: (1) every pharmacist a prescriber of care (including lifestyle modification and modifiable risk factors); and (2) every patient is seen by a pharmacist that can prescribe, if needed. The participants expressed the need for pharmacist care rendered to be consistent across inpatient and ambulatory settings. Moreover, a set of core measures could be implemented once shared understanding of the definitions of care, through creation of a data dictionary, could be achieved. Finally, a set of distribution and production core measures could be standardized across sites to foster harmonization related to product preparation and distribution.

One participant observed that, “I can’t translate the term, ‘intervention,’ into patient outcomes because I don’t follow up on recommendations.” The idea that an intervention was something that clinical pharmacy performed on another provider’s order, and not necessarily as a component of direct patient care was a major learning during this portion of the colloquium. In addition, participants expressed that their measurement of ‘intervention cost savings’ may not actually indicative of real money savings (i.e., were not green dollars), and valuation of the intervention was not standard across sites. Participants demonstrated through their own practice that “it’s all about the patient.” Finally, when given a choice to attempt to reform the concept of an ‘intervention’ into a outcomes-based revenue-generating activity or to start from scratch (or tabula rasa), as one commented, the group choose to start again and transform their care practices within the framework of what the patient needed them to do clinically. This thought process rejected the idea of trying to cost analyze the value of interventions.

In addition to setting clinical pharmacy (cp) core and custom measures (CCMs) or key performance indicators (cpKPIs), establishment of distribution and production key indicators (dpCCMs or dpKPIs) is vital to understanding the product side of the pharmacy enterprise, regardless of setting. These indicators can be based on the concept of doses dispensed or dose volume dispensed, in the case of liquid enteral and parenteral doses. Many participants expressed the need to understand how to project staffing needs from activity-based RVUs. Aligning pharmacy performance measures with MOH values and priorities will be essential to pharmacy growth, sustainment, and improvement.

*Actions to continue*

- *Refinement of pharmacy practice mission, vision, and values statements that align with MOH expectations*
- *Definition of pharmacy practice care provision in terms of the patient, and not the pharmacist or unit of practice*
- *Continuation of participation in the Federation Internationale Pharmaceutique change project at http://fip.org/ChangeTheWorld*
- *Determine the nature of the validation for advanced practice—portfolio versus objective structured clinical encounter (OSCE)*

*Actions to start*

- *Creation of Clinical Pharmacy Core and Custom Measures (cpCCMs) and Distribution and Production Core and Custom Measures (dpCCMs) through establishment of some sort of pharmacy practice parliament (PPP)*
- *Creation of data dictionary for clinical and distribution/production practice in concert with NHIPS and the Pharmaceutical Society of Singapore*
- *Measure ‘cost to dispense’ and ‘cost to advise’ within each of the three clusters and nationally in order to foster benchmarking*
- *Utilize licensure and reimbursement standards to reinforce practice change realities*

*Action to evaulate*

- *Particularizing every practice or section’s measurement activities in such a way as to maximize “uniqueness” and minimize universality*
- *Designing standards that relate only to pharmacy and not to prescribing, in general, and patient outcomes from that prescribing decision—Could you elaborate?*
- *Creation and maintenance of ‘shadow charts’ (notebooks, flowsheets, etc.) that pharmacy generates which are separate and distinct from the EHR*

## 4. Recommendations to Improve the Standard of Clinical Pharmacy and Administration in Singapore

During the last day, a continuous workshop, described earlier, was presented whose outcome was to generate a business plan for a new service, complete with performance measures, a rudimentary dashboard, and an appreciation of the usefulness of predictive analytics. The larger group was divided into four sub-groups, and assigned the task of creating this plan with the following elements: (a) purpose of the business; (b) objectives; (c) gaps that indicate need for service; (d) specific service objectives and goals; (e) customers for the new service; and (f) revenue streams for the new service. One specific new service was identified in each of the four groups: (1) diabetes management in the polyclinic; (2) understanding the environment of the dermatology patient; (3) one patient, one pharmacist philosophy applied in the ambulatory setting; and (4) “how far would you travel to see a pharmacist” or a global marketing strategy for all patients. Two root ideas were discovered in these discussions that require further development: (1) team-based primary care; and (2) community-based ambulatory care. These two models of care were identified as requiring interaction with the broader pharmacy and medical communities in order to clearly delineate the structure and function of each. The project that seemed to be the most comprehensive and developed was the insertion of a clinical pharmacist in the polyclinic environment for the purpose of improving diabetes pharmacotherapy.

*Actions to continue*

- *Regular pharmacy leadership meetings that include representation from point-of-care pharmacists and pharmacy technician leaders*

*Actions to start*

- *Develop each of the four proposals after convening a core measures group that validates measures within a Singaporean context*
- *Develop the two identified models for practice change implementation*
- *Team-based primary care (i.e., pharmacists and patient-focused pharmacy technicians) involvement in multidisciplinary teams in primary care*
- *Community-based ambulatory care (i.e., a 1 patient, 1 pharmacist philosophy)*
- *Publish methodology and findings in international pharmacy journal or book related to colloquium*
- *Prioritize money to pharmacy services that demonstrate with a clear return on investment, i.e., calculation of social, clinical, and economic return*

*Actions to evaulate*

- *Any action related to expense reduction that would ultimately devalue pharmacy services and create financially unsustainable models of care*
- *Any action related to the identification of marginal cost and benefit from pharmacist inclusion on a health team but rather, identify the value of the health team as a whole.*

## 5. Any Other Observations or Recommendations Noted during the Visit

Other observations discovered during the five-day seminar are presented in the form of manuscripts from lectures given that comprise this Special Issue called, “Proceedings of the Singapore National Clinical Pharmacy Colloquium—Health Manpower Development Plan”. This series of 10 articles will be published jointly through the MOH Office of the Chief Pharmacist, and copyright is conveyed to the MOH for this purpose.

Singaporean pharmacy has a unique opportunity to demonstrate globally as well as internally to other health care providers and countrypersons that pharmacy is on the leading edge of practice change, world-wide, toward full-scope, trans-disciplinary practice. The lecture series is arranged as follows:

Lecture 1—Justification of the value of Clinical Pharmacy services and clinical indicators measurements—Introductory remarks from a fellow traveler on a 40-year wayfaring journey within clinical pharmacy and pharmaceutical care

Lecture 2—What is a formulary, anyway? Or the Cliff Notes version of drug stewardship and expense control. HINT: there is no control

Lecture 3—Formulary nuts and bolts

Lecture 4—Capturing pharmacy’s workload in the 21st century or “we’ve got to do what we say, and say what we mean, ‘cause one thing leads us to another….”

Lecture 5—Application of Metrics to Dashboards and KPIs (clinical and operational)

Lecture 6—Predictive data analytics in Pharmacy to improve patient care: How to get there by starting where you are
